# The Alcohol Use Disorders Identification Test for Consumption (AUDIT-C) is more useful than pre-existing laboratory tests for predicting hazardous drinking: a cross-sectional study

**DOI:** 10.1186/s12889-016-3053-6

**Published:** 2016-05-10

**Authors:** Hideki Fujii, Naoki Nishimoto, Seiko Yamaguchi, Osamu Kurai, Masato Miyano, Wataru Ueda, Hiroko Oba, Tetsuya Aoki, Norifumi Kawada, Kiyotaka Okawa

**Affiliations:** Department of Gastroenterology and Hepatology, Osaka City Juso Hospital, Nonaka-kita, Yodogawa, Osaka, 532-0034 Japan; Department of Radiological Technology, Hokkaido University of Science, Maeda, Teine, Sapporo, Hokkaido 006-8585 Japan; Department of Hepatology, Graduate School of Medicine, Osaka City University, Abeno, Osaka, 545-8585 Japan

**Keywords:** Alcohol Use Disorders Identification Test, Screening, Hazardous drinking

## Abstract

**Background:**

It is important to screen for alcohol consumption and drinking customs in a standardized manner. The aim of this study was 1) to investigate whether the AUDIT score is useful for predicting hazardous drinking using optimal cutoff scores and 2) to use multivariate analysis to evaluate whether the AUDIT score was more useful than pre-existing laboratory tests for predicting hazardous drinking.

**Methods:**

A cross-sectional study using the Alcohol Use Disorders Identification Test (AUDIT) was conducted in 334 outpatients who consulted our internal medicine department. The patients completed self-reported questionnaires and underwent a diagnostic interview, physical examination, and laboratory testing.

**Results:**

Forty (23 %) male patients reported daily alcohol consumption ≥ 40 g, and 16 (10 %) female patients reported consumption ≥ 20 g. The optimal cutoff values of hazardous drinking were calculated using a 10-fold cross validation, resulting in an optimal AUDIT score cutoff of 8.2, with a sensitivity of 95.5 %, specificity of 87.0 %, false positive rate of 13.0 %, false negative rate of 4.5 %, and area under the receiver operating characteristic curve of 0.97. Multivariate analysis revealed that the most popular short version of the AUDIT consisting solely of its three consumption items (AUDIT-C) and patient sex were significantly associated with hazardous drinking. The aspartate transaminase (AST)/alanine transaminase (ALT) ratio and mean corpuscular volume (MCV) were weakly significant.

**Conclusions:**

This study showed that the AUDIT score and particularly the AUDIT-C score were more useful than the AST/ALT ratio and MCV for predicting hazardous drinking.

**Electronic supplementary material:**

The online version of this article (doi:10.1186/s12889-016-3053-6) contains supplementary material, which is available to authorized users.

## Background

Alcohol is implicated in a wide variety of diseases, disorders, and injuries, as well as social and legal problems [1, 2]. There are many forms of excessive drinking that create substantial risk or cause harm to the individual, including severe disorders such as alcohol use disorder (AUD), as well as less severe disorders such as hazardous and harmful drinking [3, 4]. In May 2013, the American Psychiatric Association issued the 5th edition of the Diagnostic and Statistical Manual of Mental Disorders (DSM–5) [5]. The DSM–5 integrates the two DSM–IV disorders, alcohol abuse and alcohol dependence, into a single disorder called AUD with mild, moderate, and severe sub-classifications [5]. Hazardous drinking, recognized by the World Health Organization (WHO), is defined as a quantity or pattern of alcohol consumption that places the individual at risk for adverse health events [3, 6]. Harmful drinking, defined as alcohol consumption that negatively affects physical and mental health, is also recognized by the WHO [3, 6]. Alcohol-related problems pose a huge economic cost to many communities worldwide. Screening and brief intervention for alcohol has emerged as a cost-effective preventative approach [7] that is relevant and practical for delivery in a primary health care setting. However, there is considerable inconsistency in the reporting and interpretation of alcohol use both by subjects and physicians across studies [8]. Therefore, it is important to screen for alcohol consumption and drinking customs using a standardized method.

Laboratory methods to screen for alcohol consumption include tests such as blood ethanol, serum γ - glutamyltransferase (GGT), carbohydrate-deficient transferrin (CDT), and mean corpuscular volume of erythrocytes (MCV), aspartate aminotransferase (AST), and alanine aminotransferase (ALT) [9]. Previously, Aertgeerts et al. reported that laboratory tests are of no use for detecting alcohol abuse or dependency in a primary care setting [10]. Meanwhile, Dunn et al. more recently reported the alcoholic liver disease (ALD)/non-alcoholic fatty liver disease (NAFLD) index (ANI) for the differentiation of ALD and NAFLD [11]. ANI is a formula derived from four independent ALD predictors: MCV, the AST/ALT ratio, body mass index (BMI), and male sex that has been reported as capable of distinguishing ALD from NAFLD [11, 12].

In addition to laboratory tests, the Alcohol Use Disorders Identification Test (AUDIT) is a 10-item questionnaire designed by the WHO to screen for hazardous drinking in primary health care settings (Additional file 1: Table S1) [13, 14]. It was developed and evaluated over two decades, and provides an accurate measure of risk across sex, age, and cultures [3, 13–15]. The Alcohol Use Disorders Identification Test for Consumption (AUDIT-C), the most popular short version of the AUDIT consisting solely of three consumption items, is approximately equal in accuracy to the full AUDIT [15–17].

Health educators and researchers in different countries employ different definitions of a standard unit or drink because of differences in the typical serving sizes in that country [18]. For example, one standard drink in the United States contains 12–14 g of pure alcohol, one standard drink in the United Kingdom contains 8–10 g, one standard drink in Australia contains 10 g, and one standard drink in Japan contains 20–23.5 g [18]. The AUDIT must be adapted for different countries due to different definitions of a standard unit or drink.

Some studies have examined the prevalence of unhealthy alcohol use in the hospital outpatient setting using AUDIT [19–22]. The prevalence of unhealthy alcohol use tended to be lower in studies conducted in general outpatient populations. Of these, three studies were conducted in general outpatients mainly in the internal medicine department. In two studies, estimates of unhealthy alcohol use ranged from 6 % in a Dutch hospital to 38 % in a South African hospital. On the other hand, one study from Japan reported the prevalence of problematic drinking or alcohol use disorders in outpatients [23]. The prevalence of “heavy drinking” and “suspected alcohol dependence” were 7.1 and 14.1 %, respectively. They concluded that screening problematic drinking is required for early detection in patients visiting the internal medicine and surgical departments of general hospitals.

Nonetheless, the optimal cutoff value of the AUDIT score to predict “hazardous drinking” in Japanese internal medicine outpatients is unknown. Moreover, it remains unclear whether both the AUDIT score and pre-existing laboratory tests are independent predictors of hazardous drinking.

Accordingly, the aim of this study was 1) to investigate whether the AUDIT score is useful for predicting hazardous drinking using optimal cutoff scores and 2) to use multivariate analysis to evaluate whether the AUDIT score was more useful than pre-existing laboratory tests for predicting hazardous drinking.

## Methods

### Design

This exploratory cross-sectional study included patients who visited the Internal Medicine Department of Osaka City Juso Hospital, Japan between June 25, 2014 and March 4, 2015.

### Patients

The internal medicine and general hepatology outpatient clinic was held once per week and attended by a single hepatologist. Participation in the study was proposed systematically to all adult (age ≥ 18 years) patients who visited this outpatient clinic. Patients were excluded if they had an acute psychotic or manic episode, had a severe major depressive episode, did not understand the aims of the study, did not complete screening for daily alcohol consumption including AUDIT, and/or did not understand Japanese. We also excluded patients who had not undergone testing for hepatitis B surface antigen (HBsAg) and hepatitis C virus (HCV) antibody in our hospital. The doctor examined 522 patients who visited our hospital during the above-mentioned period. Of these, seven were excluded because the AUDIT was not performed (due to depressed levels of consciousness [*n* = 2] and family consultation [*n* = 5]). Thus, 515 patients were assessed. One hundred eighty-one patients were excluded because of a lack of data for HBsAg and/or HCV antibody. The remaining 334 patients were included in this study. The study protocol was approved by the Institutional Review Board at Osaka City Juso Hospital, and the participants provided informed consent. The study was conducted in accordance with the Declaration of Helsinki.

### Alcohol screening measures

Each patient underwent a comprehensive assessment of the presenting disorder as well as daily alcohol consumption, including AUDIT. We assessed drinking frequency (daily, weekly, monthly, and yearly) along with the volume of alcohol intake according to beverage types. The volume was subsequently converted to grams of ethanol, and values for each beverage type were added. The ethanol contents for calculation were as follows: 5 % for beer, 25 % for shochu (a distilled alcoholic beverage made in Japan), 15 % for Japanese sake (rice wine), 43 % for whisky, and 14 % for wine. The specific density of alcohol was defined as 0.79 g/mL [8]. We developed an automatic calculator to determine the amount of alcohol in a typical drink for electronic medical records using a template (Juso Alcohol Calculator; JAC), and entered the data therein (Additional file 1: Table S2).

The AUDIT was administered in combination with an oral interview and a self-reported questionnaire. The patients responded to the questionnaire in the waiting room while they waited for their medical visit. The patients were subsequently interviewed by the doctor to clarify ambiguous answers. In this study, we used the Japanese version of the AUDIT, which defines one standard drink as 20 g of pure alcohol in accordance with current available health guidance in Japan. The doctor provided this information to patients using the Standard Drink chart. Response options on the AUDIT [13, 14] assess the frequency of a particular drinking- related behavior over the preceding year. AUDIT scores range from 0 to 40 and are calculated by summing the scores for all 10 items. All subjects completed the AUDIT in combination with a self-report questionnaire and oral interview (Additional file 1: Table S1). Hazardous drinking was defined as the modified criteria employed by Bohn et al. [24] : daily ethanol consumption ≥ 40 g for male or ≥ 20 g for females.

### Anthropometric and laboratory evaluation

Anthropometric variables (height and weight) were measured using a calibrated scale after requesting that the patients remove their shoes and any heavy clothing. BMI was calculated as weight (kg) divided by the square of height in meters (m^2^). Obesity was defined as a BMI of > 25 kg/m^2^ according to the Japanese criteria for obesity [25]. Patients were assigned a diagnosis of diabetes mellitus if they had documented use of oral hypoglycemic medication, a random glucose level > 200 mg/dl, or fasting plasma glucose > 126 mg/dl [26]. Dyslipidemia was diagnosed if the cholesterol level was > 220 mg/dl and/or the triglyceride level was > 150 mg/dl. Hypertension was diagnosed if the patient was taking antihypertensive medication and/or had a resting recumbent blood pressure ≥ 140/90 mmHg on at least two occasions. Venous blood samples were obtained in the morning after the patients had fasted overnight for 12 h. Laboratory evaluations for all patients included determination of platelet counts and measurement of the serum levels of AST, ALT, the AST/ALT ratio, the serum levels of GGT, and MCV. HBsAg was measured using the ARCHITECT HBsAg QT assay (Abbott Japan, Tokyo, Japan), and anti-HCV antibodies were measured using the ARCHITECT HCV QT assay (Abbott Japan). All parameters were measured using standard techniques.

### Statistical analysis

Statistical analysis was conducted using JMP 10.0 software (SAS Institute Inc. Cary, NC). Continuous variables were expressed as mean ± standard deviation. Qualitative data were represented as numbers, with the percentages indicated in parentheses. Sensitivity and specificity, which reflect the probabilities of false positive and false negative assessment, respectively, were determined for selected cutoff values, and the area under the receiver operating characteristic curve (AUROC) was calculated. The Youden index was used to identify the optimal cutoff points. We used 10-fold cross-validation to check internal validity. Briefly, in this method the original sample is randomly partitioned into 10 approximately equally sized subsamples. Of the 10 subsamples, a single subsample is retained as the validation data for testing the model, and the remaining nine subsamples are used as training data. The cross-validation process is then repeated 10 times, with each of the 10 subsamples used exactly once as the validation data. The 10 subsamples results can then be averaged (or otherwise combined) to produce a single estimation. Multivariate logistic regression analyses were conducted to identify parameters that significantly contributed to the estimation of hazardous drinking. Differences with *P* values <0.05 were considered statistically significant.

## Results

### Patient characteristics

Patient clinical characteristics are summarized in Table 1. One hundred and sixty patients (48 %) were female, and 126 patients (38 %) were obese. The ratios of metabolic factors were between 20 and 30 %. The mean AST/ALT ratio was > 1 in both male and female patients. Eighteen patients (5.4 %) were positive for HBsAg, and 42 patients (12.6 %) were positive for HCV antibody. The prevalence of disorders in the cohort is shown in Table 2. One hundred and sixty-two patients (48.5 %) were diagnosed with hepatic and biliary disorders, 58 (17.4 %) with infectious disorders, 31 (9.3 %) with endocrine and metabolic disorders, 14 (4.1 %) with cardiovascular disorders, nine (2.7 %) with neurological disorders, and seven (2.1 %) with gastrointestinal disorders. Furthermore, 10.2 % of patients were diagnosed as having an unclassified disorder.

**Table 1:** Patient characteristics

	Male (*N* = 174)	Female (*N* = 160)
Age (years) ^a^	58.4 (16.2)	56.8 (17.6)
BMI (kg/m^2^) ^a^	25.1 (4.7)	23.5 (4.7)
Diabetes^b^	35 (21.0 %)	28 (21.4 %)
Hypertension^b^	36 (21.6 %)	27 (20.6 %)
Dyslipidemia^b^	46 (27.9 %)	34 (27.0 %)
AST (IU/l) ^c^	29 (12–209)	24 (9–1165)
ALT (IU/L) ^c^	28 (6–226)	19 (6–1573)
AST/ALT ratio^a^	1.21 (0.6)	1.36 (0.6)
GGT (IU/L) ^c^	56.5 (10–1932)	25 (8–588)
MCV (fl) ^a^	89.9 (8.3)	88.8 (5.1)
HBsAg^b^	10 (5.7 %)	8 (5.0 %)
HCV-Ab^b^	22 (12.6 %)	20 (12.5 %)

^a^Mean (SD), ^b^number (%), ^c^median (range). *AST* aspartate transaminase, *ALT* alanine transaminase, *BMI* body mass index, *GGT* γ-glutamyltransferase, *HBsAg* hepatitis B surface antigen, *HCV-Ab* hepatitis C virus-antibody, *MCV* mean corpuscular volume

**Table 2:** Prevalence of patient disorders

Hepatic and biliary	162 (48.5 %)
Infectious	58 (17.4 %)
Endocrine and metabolic	31 (9.3 %)
Cardiovascular	14 (4.1 %)
Neurological	9 (2.7 %)
Gastrointestinal	7 (2.1 %)
Hematology and oncology	4 (1.2 %)
Pulmonary	4 (1.2 %)
Ear, nose, and throat	3 (0.9 %)
Musculoskeletal and connective tissue	3 (0.9 %)
Genitourinary	2 (0.6 %)
Psychiatric	2 (0.6 %)
Nutritional	1 (0.3 %)
Unclassified	34 (10.2 %)
Total	334 (100 %)

### Relationship between patients’ alcohol consumption and AUDIT scores

Table 3 shows the prevalence of alcohol consumption and AUDIT scores. Forty male patients (23 %) had daily alcohol consumption ≥ 40 g, and 16 female patients (10 %) consumed ≥ 20 g daily. At the same time, 69 male patients (39 %) and 16 female patients (10 %) had an AUDIT score ≥ 8.

**Table 3:** Prevalence of alcohol consumption and AUDIT score

	Male (*N* = 174)	Female (*N* = 160)
Alcohol consumption (g/day)		
<20	107 (61.5 %)	144 (90.0 %)
20–39.9	27 (15.5 %)	9 (5.6 %)
40–59.9	13 (7.5 %)	3 (1.8 %)
60–79.9	16 (9.2 %)	2 (1.3 %)
80–99.9	4 (2.3 %)	0 (0 %)
≥100	7 (4.0 %)	2 (1.3 %)
AUDIT score		
<5	80 (46.0 %)	132 (82.5 %)
5–9	43 (24.7 %)	19 (11.8 %)
10–14	19 (16.7 %)	3 (1.8 %)
15–19	11 (6.3 %)	2 (1.3 %)
20–24	8 (4.6 %)	2 (1.3 %)
≥25	3 (1.7 %)	2 (1.3 %)

### Appropriate cutoffs of AUDIT scores

For 10-fold cross-validation, mean values of sensitivity, specificity, false positive, false negative, and area under the receiver operating characteristic curve (AUROC) for hazardous drinking are shown in Table 4. For hazardous drinking, the optimal cutoff for the AUDIT score was 8.2.

**Table 4:** AUDIT score optimal cutoff value statistics

	AUDIT score	Se	Sp	FP	FN	AUROC
Alcohol consumption (g/day)						
Male^a^ ≥ 40 of Female^b^ ≥ 20	8.2	95.5	87.0	13.0	4.5	0.97

^a^ *N* = 174, ^b^ *N* = 160. *Se* sensitivity, *Sp* specificity, *FN* false negative, *FP* false positive, *AUROC* area under the receiver operating characteristic curve

### Sensitivity and specificity of the optimal cutoff value of the AUDIT score

The sensitivity and specificity of an AUDIT score ≥ 8 men were 97.5 and 79.1 %, and for a score ≥ 4 women were 100 and 85.4 %, respectively. The sensitivity and specificity of an AUDIT score ≥ 5 in all patients were 100 and 76.3 %, respectively. With a cutoff value for AUDIT score of ≥ 5, almost 40 % of male patients were misdiagnosed (Table 5).

**Table 5:** Sensitivity and specificity of optimal AUDIT cutoff scores

	Men^a^		Women^b^		Total	
	Se	Sp	Se	Sp	Se	Sp
AUDIT ≥ 8 men ≥ 4 women	97.5	79.1	100	85.4		
AUDIT ≥ 5	100	59.7	100	91.7	100	76.3

^a^ *N* = 174, ^b^ *N* = 160. *Se* sensitivity, *Sp* specificity

### Sensitivity and specificity of the optimal cutoff value of other variables

The sensitivity and specificity of BMI ≥ 27.7 were 30.4 and 78.7 %, AST/ALT ratio ≥ 1.2 were 57.7 and 60.6 %, GGT ≥ 80 were 58.0 and 81.5 %, and MCV ≥ 93 were 53.8 and 75.9 %, respectively (Table 6).

**Table 6:** Sensitivity and specificity of optimal cutoff values of other variables

Hazardous drinking (g/day)	Cut-off values	Se	Sp
Male^a^ ≥40, Female^b^ ≥20			
BMI	27.7	30.4	78.7
AST/ALT ratio	1.2	57.7	60.6
GGT	80	58.0	81.5
MCV	93	53.8	75.9

^a^ *N* = 174, ^b^ *N* = 160, *Se* sensitivity, *Sp* specificity

### Logistic regression analysis

On univariate analysis for baseline predictors, Question 1, Question 2 and Question 3 were significantly related with an alcohol consumption ≥ 40 g per day in male patients and ≥ 20 g per day in female patients (all *P* < 0.001; Table 7). Multivariate analysis identified only AUDIT-C and patient sex as significant independent predictors of hazardous drinking. The AST/ALT ratio and MCV were only weakly significant (Table 8).

**Table 7:** Univariate analysis for the prediction of hazardous drinking

Hazardous drinking (g/day)	OR	95%CI	*P* value
Male^a^ ≥ 40			
Question 1	5.03	2.53–10.01	<0.001
Question 2	4.20	2.73–6.46	<0.001
Question 3	5.70	3.32–9.79	<0.001
Female^b^ ≥ 20			
Question 1	5.14	2.49–10.60	<0.001
Question 2	7.69	3.43–17.24	<0.001
Question 3	7.07	3.52–14.20	<0.001

^a^ *N* = 174, ^b^ *N* = 160, *OR* odds ratio, *CI* confidence interval

**Table 8:** Multivariate analysis for the prediction of hazardous drinking

Hazardous drinking (g/day) Male^a^ ≥ 40, Female^b^ ≥ 20	OR	95%CI	*P* value
AUDIT-C	3.45	2.26–5.26	<0.001
Sex (Female)	8.92	1.67–47.6	0.010
BMI	1.08	0.95–1.24	0.248
AST/ALT ratio	2.94	0.95–9.12	0.063
GGT	1.00	1.00–1.01	0.895
MCV	1.10	0.99–1.21	0.084

^a^ *N* = 174, ^b^ *N* = 160, *OR* odds ratio, *CI* confidence interval

## Discussion

The AUDIT score is useful for predicting hazardous drinking by using optimal cutoff values. Since the AUDIT was published, a number of studies have evaluated its validity and reliability in different clinical and community samples worldwide [3, 13–15]. At the recommended cutoff of 8, most studies have found very favorable sensitivity and usually lower, but still acceptable, specificity, for current ICD-10 alcohol use disorders [15]. The present study showed that using an optimal AUDIT cutoff score of 8.2 resulted in a high sensitivity of 95.5 % and a high specificity of 87 % for identifying hazardous drinkers among outpatients at an internal medicine department. The biologically effective dose of alcohol in relation to mortality in female patients is approximately two standard drinks per day less than that of male patients [27].

Therefore, we first evaluated sex-specific AUDIT cutoff scores for hazardous drinking separately. Among male patients, the optimal cutoff score of 10 resulted in a decrease in sensitivity from 95.8 to 94.2 % and an increase in specificity from 87.0 to 90.1 %. Among female patients, an optimal cutoff score of 6.1 resulted in a decrease in sensitivity from 95.8 to 88.9 % and an increase in specificity from 87.0 to 93.4 %. Reinert et al. suggested that optimal cutoff value of AUDIT score is lower in women than in men [15, 29]. In addition, the 2005 edition of the National Institute on Alcohol Abuse and Alcoholism Guide selected an optimal cutoff of eight for men and four for women [28]. In contrast, it has been reported that an AUDIT score cutoff of five may be appropriate for women [29]. Second, we evaluated the sensitivity and specificity of an AUDIT score ≥ 8 in men, ≥ 4 in women, ≥ and 5 in all patients (Table 5). As shown in Table 4, the sensitivity and specificity were relatively unchanged. With an AUDIT cutoff value of ≥ 5, specificity decreased to 59.7 % in men. In women, there was a limit to the accuracy of estimation because only a small number of women were classified with hazardous drinking as compared with men. Although it is difficult to conclusively establish because of a different methods of analysis, our study suggested an appropriate cutoff value of eight for hazardous drinking in Japanese internal medicine outpatients.

We also evaluated whether the AUDIT questionnaire was more useful than pre-existing laboratory tests for predicting hazardous drinking. After adjusting for BMI, the AST/ALT ratio, GGT, and MCV, the AUDIT-C and patient sex were significantly associated with hazardous drinking. Furthermore, univariate analysis revealed that only Questions 1, 2, and 3 were statistically significantly associated with hazardous drinking (Table 7). Multicollinearity is thought to strongly affect to these questions because they are similar quantity frequency assessments. Therefore, to avoid multicollinearity, we used the AUDIT-C in subsequent multivariate analysis. After taking into consideration that hazardous drinking is defined differently for male and female patients, female sex had a significant odds ratio for hazardous drinking. One plausible explanation for this finding is that the estimate of the sex effect was unstable because of multicollinearity with AUDIT-C scores.

Our data suggested that the AST/ALT ratio and MCV had borderline significance in predicting hazardous drinking. The AST/ALT ratio appeared to be a useful index to distinguish non-alcoholic steatohepatitis and ALD [30], and a high AST/ALT ratio suggested advanced ALD [31]. Furthermore, MCV is often used as part of the screening procedures for the detection of alcohol abuse [20]. Bohn et al. reported that the AUDIT score demonstrated significant, moderate, positive correlations with MCV [24]. Further large scale multicenter studies are needed to clarify whether the AST/ALT ratio and/or MCV are useful predictors for the early stages of ALD.

A recent study validated the Japanese version of the AUDIT [32]. This study revealed that 8.0 % of their sample had an AUDIT score ≥ 11 (indicating alcohol abuse). In this study, the 145 subjects (136 men) who answered the questionnaire worked in a car manufacturing company. However, there were no details about alcohol consumption [such as drinking frequency (daily, weekly, monthly, or yearly) or volume of alcohol intake according to beverage type]. In our study, 53 patients (16 %) had an AUDIT score ≥ 11. Of these, 42 patients drank four or more times per week, 26 patients drank seven or more drinks on a typical day, and 29 patients had six or more drinks daily or almost daily. Additionally, we demonstrated the internal validity of our data by using 10-fold cross-validation.

We evaluated the effect of alcohol consumption on pre-existing laboratory tests excluding the possibility of hepatitis B (HBV) and HCV infection. Chronic infection with HBV or HCV is the major cause of end-stage liver disease including cirrhosis and hepatocellular carcinoma [33, 34], and the coexistence of these factors have a synergistic hepatotoxic effect, and increase the risk of advanced liver disease [35–37]. In this study, the AST/ALT ratio was significantly higher in HBV- or HCV- positive patients than in those patients who were virus negative. Additionally, the platelet count was significantly lower in virus positive patients than in those who were virus negative (data not shown). Based on these results, we speculate that the stage of liver fibrosis was more severe in virus positive patients than in those who were negative. Regardless, even when excluding HBsAg- and HCV antibody- positive patients, the optimal cutoff values for hazardous drinking was still 8.2.

The main strengths of this study are that the data concerning alcohol consumption and drinking customs were screened by one doctor using a standardized method. Using the JAC, by only entering the digit of frequency, kind, and amount, a doctor can accurately determine the amount of daily alcohol consumption, and report the data to the patients immediately. The standard drink concept suggests that there is a serving size of alcohol that is typical for a particular country. Thus, the use of the standard drink it is complicated by different standards across and even within countries [14]. To prevent confusion, we used the Japanese version of the Standard Drink chart, which shows the “unit” and the “number of drinks” in parallel.

Furthermore, we established internal validity by using 10-fold cross-validation. Using multivariate analysis, we also evaluated whether the AUDIT score was more useful than pre-existing laboratory tests for predicting hazardous drinking.

Some limitations to this study should also be noted. First, external validity was not investigated because this study comprised exploratory research. Second, the rate of hepatic and biliary disorders tended to be high because the doctor was a hepatologist. The doctor screened all patients who visited his outpatient department to avoid patient selection bias. Third, we lacked adequate patient information regarding psychosocial characteristics such as the Structured Clinical Interview for DSM-IV, Axes I Disorders. Underestimation of alcohol use disorders may have serious consequences in the mental health setting [38]. Although two patients in stable condition who had chronic psychiatric disease were excluded, it is possible that potential psychiatric patients were still included (Table 2). Future prospective studies should validate the AUDIT for the early identification of psychiatric patients relative to internal medicine outpatients.

## Conclusions

The AUDIT score is useful for predicting hazardous drinking with an optimal cutoff in outpatients visiting a Japanese internal medicine department. The AUDIT-C score and patient sex were significantly associated with hazardous drinking, and the AST/ALT ratio and MCV were weakly significant.

## Ethics approval and consent to participate

The study protocol was approved by the Institutional Review Board at Osaka City Juso Hospital, and the participants provided informed consent. The study was conducted in accordance with the Declaration of Helsinki.

## Additional file

Additional file 1:
**Table S1.** Alcohol Use Disorders Identification Test; the standardized questionnaire used to assess hazardous drinking. **Table S2.** Juso Alcohol Calculator (JAC); the method used to calculate alcohol consumption and grams based on patients’ reported alcohol consumption volume and type. (PPTX 79 kb)

## Abbreviations

ALD: alcoholic liver disease
ALT: alanine transaminase
ANI: ALD/NAFLD index
AST: aspartate transaminase
AUD: alcohol use disorder
AUDIT: Alcohol Use Disorders Identification Test
AUROC: area under the receiver operating characteristic curve
BMI: body mass index
GGT: γ-glutamyl transferase
HBsAg: hepatitis B surface antigen
HBV: hepatitis B virus
HCV: hepatitis C virus
JAC: Juso Alcohol Calculator
MCV: mean corpuscular volume
NAFLD: non-alcoholic fatty liver disease
WHO: World Health Organization

## References

[1] Connor JP, Haber PS, Hall WD (2015). Alcohol use disorders. The Lancet.

[2] Rehm J, Mathers C, Popova S, Thavorncharoensap M, Teerawattananon Y, Patra J (2009). Global burden of disease and injury and economic cost attributable to alcohol use and alcohol-use disorders. The Lancet.

[3] Fiellin DA, Reid MC, O'Connor PG (2000). Screening for alcohol problems in primary care: a systematic review. Arch Intern Med.

[4] Saunders JB, Lee NK (2000). Hazardous alcohol use: its delineation as a subthreshold disorder, and approaches to its diagnosis and management. Compr Psychiatry.

[5] Rinker DV, Neighbors C (2015). Latent Class Analysis of DSM-5 Alcohol Use Disorder Criteria Among Heavy-Drinking College Students. J Subst Abuse Treat..

[6] Rehm J, Anderson P, Manthey J, Shield KD, Struzzo P, Wojnar M, Gual A. Alcohol Use Disorders in Primary Health Care: What Do We Know and Where Do We Go? Alcohol Alcohol*.* 2015 Nov 15. pii: abv127. [Equip ahead of print]. 10.1093/alcalc/agv127 26574600

[7] Hutubessy R, Chisholm D, Edejer TT (2003). Generalized cost-effectiveness analysis for national-level priority-setting in the health sector. Cost Eff Resour Alloc.

[8] Brick J (2006). Standardization of alcohol calculations in research. Alcohol Clin Exp Res.

[9] Niemelä O (2007). Biomarkers in alcoholism. Clin Chim Acta.

[10] Aertgeerts B, Buntinx F, Ansoms S, Fevery J (2001). Screening properties of questionnaires and laboratory tests for the detection of alcohol abuse or dependence in a general practice population. Br J Gen Pract.

[11] Dunn W, Angulo P, Sanderson S, Jamil LH, Stadheim L, Rosen C, Malinchoc M, Kamath PS, Shah VH (2006). Utility of a new model to diagnose an alcohol basis for steatohepatitis. Gastroenterology.

[12] Cerović I, Mladenović D, Ješić R, Naumović T, Branković M, Vučević D, Aleksić V, Radosavljević T (2013). Alcoholic liver disease/nonalcoholic fatty liver disease index: distinguishing alcoholic from nonalcoholic fatty liver disease. Eur J Gastroenterol Hepatol.

[13] Burns E, Gray R, Smith LA (2010). Brief screening questionnaires to identify problem drinking during pregnancy: a systematic review. Addiction.

[14] de Oliveira JB, Kerr-Corrêa F, Lima MC, Bertolote JM, Santos JL (2014). Validity of alcohol screening instruments in general population gender studies: an analytical review. Curr Drug Abuse Rev.

[15] Reinert DF, Allen JP (2007). The alcohol use disorders identification test: an update of research findings. Alcohol Clin Exp Res.

[16] Aertgeerts B, Buntinx F, Bande-Knops J, Vandermeulen C, Roelamts M, Ansoms S, Fevery J (2000). The value of CAGE, CUGE, and AUDIT in screening for alcohol abuse and dependence among college freshmen. Alcohol Clin Exp Res.

[17] Gordon AJ, Maisto SA, McNeil M, Kraemer KL, Conigliaro RL, Kelley ME, Conigliaro J (2001). Three questions can detect hazardous drinkers. J Fam Pract.

[18] Greenfield TK, Kerr WC (2008). Alcohol measurement methodology in epidemiology: recent advances and opportunities. Addiction.

[19] Emmen MJ, Schippers GM, Wollersheim H, Bleijenberg G (2005). Adding psychologist's intervention to physicians' advice to problem drinkers in the outpatient clinic. Alcohol Alcohol.

[20] Pengpid S, Peltzer K, Van der Heever H (2011). Prevalence of alcohol use and associated factors in urban hospital outpatients in South Africa. Int J Environ Res Public Health.

[21] Chang G, Fisher ND, Hornstein MD, Jones JA, Hauke SH, Niamkey N, Briegleb C, Orav EJ (2011). Brief intervention for women with risky drinking and medical diagnoses: a randomized controlled trial. J Subst Abuse Treat..

[22] Johnson NA, Kypri K, Latter J, McElduff P, Saunders JB, Saitz R, Attia J, Dunlop A, Doran C, Wolfenden L (2014). Prevalence of unhealthy alcohol use in hospital outpatients. Drug Alcohol Depend..

[23] Akazawa M, Matsumoto T, Kumagai N (2013). Prevalence of problematic drinking among outpatients attending general hospitals in Tokyo. Nihon Arukoru Yakubutsu Igakkai Zasshi.

[24] Bohn MJ, Babor TF, Kranzler HR (1995). The Alcohol Use Disorders Identification Test (AUDIT): validation of a screening instrument for use in medical settings. J Stud Alcohol.

[25] The Examination Committee of Criteria for ‘Obesirt Disease’ in Japan; Japan Society for the Study of Obesity (2002). New criteria for 'obesity disease' in Japan. Circ J.

[26] Expert Committee on the Diagnosis and Classification of Diabetes Mellitus (2003). Report of the expert committee on the diagnosis and classification of diabetes mellitus. Diabetes Care.

[27] Rehm J, Room R, Monteiro M, Gmel G, Graham K, Rehn N, Sempos CT, Jernigan D (2003). Alcohol as a risk factor for global burden of disease. Eur Addict Res.

[28] National Institute on Alcohol Abuse and Alcoholism and National Institute of Health. Helping Patients Who Drink Too Much. 2005. Department of Health and Human Services, Public Health Service, National Institutes of Health. http://pubs.niaaa.nih.gov/publications/Practitioner/CliniciansGuide2005/guide.pdf.

[29] Reinert DF, Allen JP (2002). The Alcohol Use Disorders Identification Test (AUDIT): a review of recent research. Alcohol Clin Exp Res.

[30] Toshikuni N, Tsutsumi M, Arisawa T (2014). Clinical differences between alcoholic liver disease and nonalcoholic fatty liver disease. World J Gastroenterol.

[31] Nyblom H, Berggren U, Balldin J, Olsson R (2004). High AST/ALT ratio may indicate advanced alcoholic liver disease rather than heavy drinking. Alcohol Alcohol.

[32] Kawada T, Inagaki H, Kuratomi Y (2011). The alcohol use disorders identification test: reliability study of the Japanese version. Alcohol.

[33] European Association For The Study Of The Liver (2012). EASL clinical practice guidelines: Management of chronic hepatitis B virus infection. J Hepatol..

[34] European Association For The Study Of The Liver (2015). EASL Recommendations on Treatment of Hepatitis C 2015. J Hepatol..

[35] Bedogni G, Miglioli L, Masutti F, Ferri S, Castiglione A, Lenzi M, Crocè LS, Granito A, Tiribelli C, Bellentani S (2008). Natural course of chronic HCV and HBV infection and role of alcohol in the general population: the Dionysos Study. Am J Gastroenterol.

[36] Gitto S, Vitale G, Villa E, Andreone P (2014). Update on Alcohol and Viral Hepatitis. J Clin Transl Hepatol.

[37] Novo-Veleiro I, Alvela-Suárez L, Chamorro AJ, González-Sarmiento R, Laso FJ, Marcos M (2016). Alcoholic liver disease and hepatitis C virus infection. World J Gastroenterol.

[38] Agabio R, Marras P, Gessa GL, Carpiniello B (2007). Alcohol use disorders, and at-risk drinking in patients affected by a mood disorder, in Cagliari, Italy: sensitivity and specificity of different questionnaires. Alcohol Alcohol.

