# Supplementary material for: The Alcohol Use Disorders Identification Test for Consumption (AUDIT-C) is more useful than pre-existing laboratory tests for predicting hazardous drinking: a cross-sectional study
Source: BMC Public Health. 2016 May 10;16:379. doi: 10.1186/s12889-016-3053-6 (PMC4862044; doi:10.1186/s12889-016-3053-6)
Supplement: Additional file 1: — Table S1. Alcohol Use Disorders Identification Test; the standardized questionnaire used to assess hazardous drinking. Table S2. Juso Alcohol Calculator (JAC); the method used to calculate alcohol consumption and grams based on patients’ reported alcohol consumption volume and type. (PPTX 79 kb) [file 12889_2016_3053_MOESM1_ESM.pptx]

## Slide 1
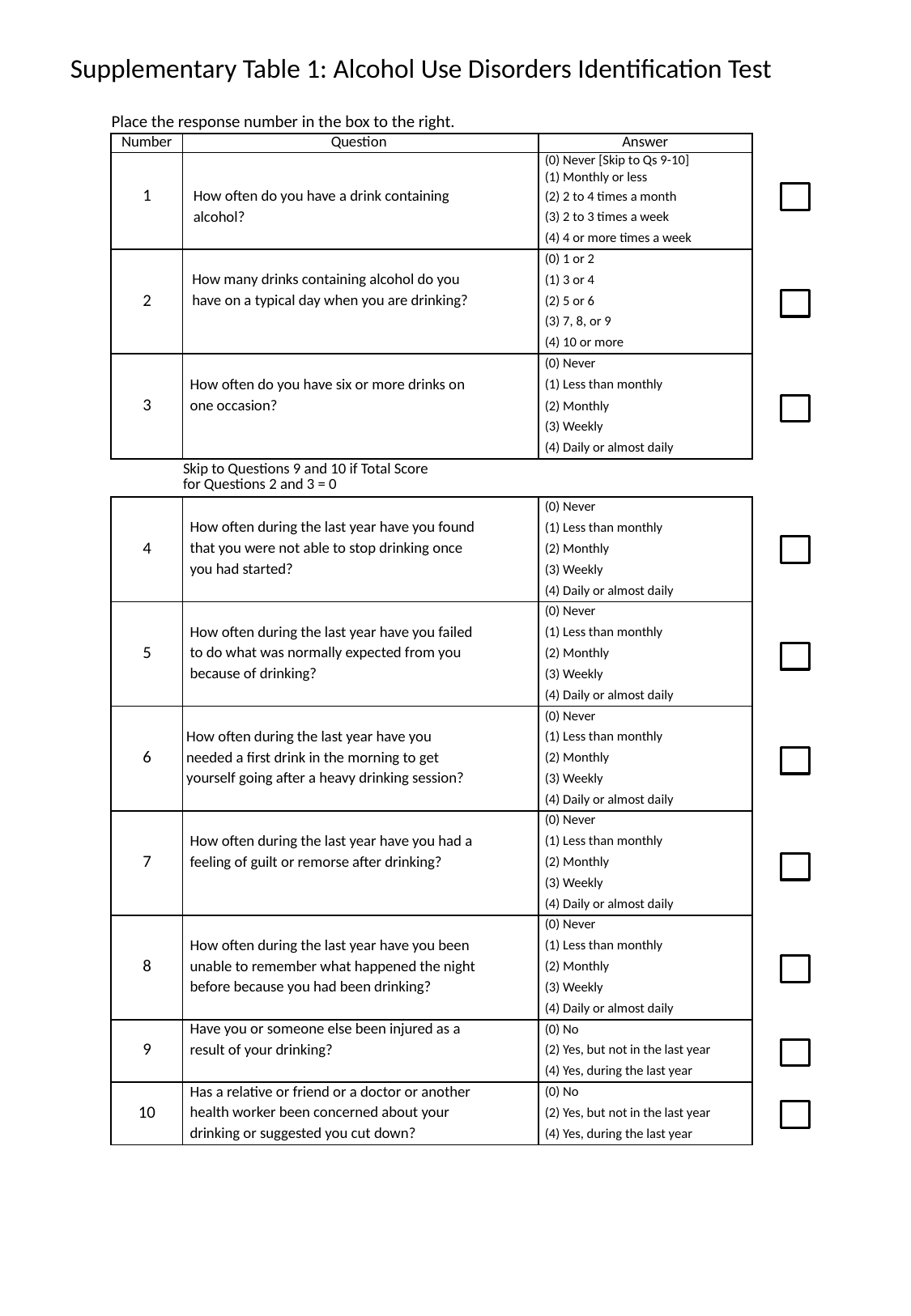

Supplementary Table 1: Alcohol Use Disorders Identification Test
| Place the response number in the box to the right. | | |
| --- | --- | --- |
| Number | Question | Answer |
| | | (0) Never [Skip to Qs 9-10] |
| | | (1) Monthly or less |
| 1 | How often do you have a drink containing | (2) 2 to 4 times a month |
| | alcohol? | (3) 2 to 3 times a week |
| | | (4) 4 or more times a week |
| | | (0) 1 or 2 |
| | How many drinks containing alcohol do you | (1) 3 or 4 |
| 2 | have on a typical day when you are drinking? | (2) 5 or 6 |
| | | (3) 7, 8, or 9 |
| | | (4) 10 or more |
| | | (0) Never |
| | How often do you have six or more drinks on | (1) Less than monthly |
| 3 | one occasion? | (2) Monthly |
| | | (3) Weekly |
| | | (4) Daily or almost daily |
| | Skip to Questions 9 and 10 if Total Score for Questions 2 and 3 = 0 | |
| | | (0) Never |
| | How often during the last year have you found | (1) Less than monthly |
| 4 | that you were not able to stop drinking once | (2) Monthly |
| | you had started? | (3) Weekly |
| | | (4) Daily or almost daily |
| | | (0) Never |
| | How often during the last year have you failed | (1) Less than monthly |
| 5 | to do what was normally expected from you | (2) Monthly |
| | because of drinking? | (3) Weekly |
| | | (4) Daily or almost daily |
| | | (0) Never |
| | How often during the last year have you | (1) Less than monthly |
| 6 | needed a first drink in the morning to get | (2) Monthly |
| | yourself going after a heavy drinking session? | (3) Weekly |
| | | (4) Daily or almost daily |
| | | (0) Never |
| | How often during the last year have you had a | (1) Less than monthly |
| 7 | feeling of guilt or remorse after drinking? | (2) Monthly |
| | | (3) Weekly |
| | | (4) Daily or almost daily |
| | | (0) Never |
| | How often during the last year have you been | (1) Less than monthly |
| 8 | unable to remember what happened the night | (2) Monthly |
| | before because you had been drinking? | (3) Weekly |
| | | (4) Daily or almost daily |
| | Have you or someone else been injured as a | (0) No |
| 9 | result of your drinking? | (2) Yes, but not in the last year |
| | | (4) Yes, during the last year |
| | Has a relative or friend or a doctor or another | (0) No |
| 10 | health worker been concerned about your | (2) Yes, but not in the last year |
| | drinking or suggested you cut down? | (4) Yes, during the last year |

## Slide 2
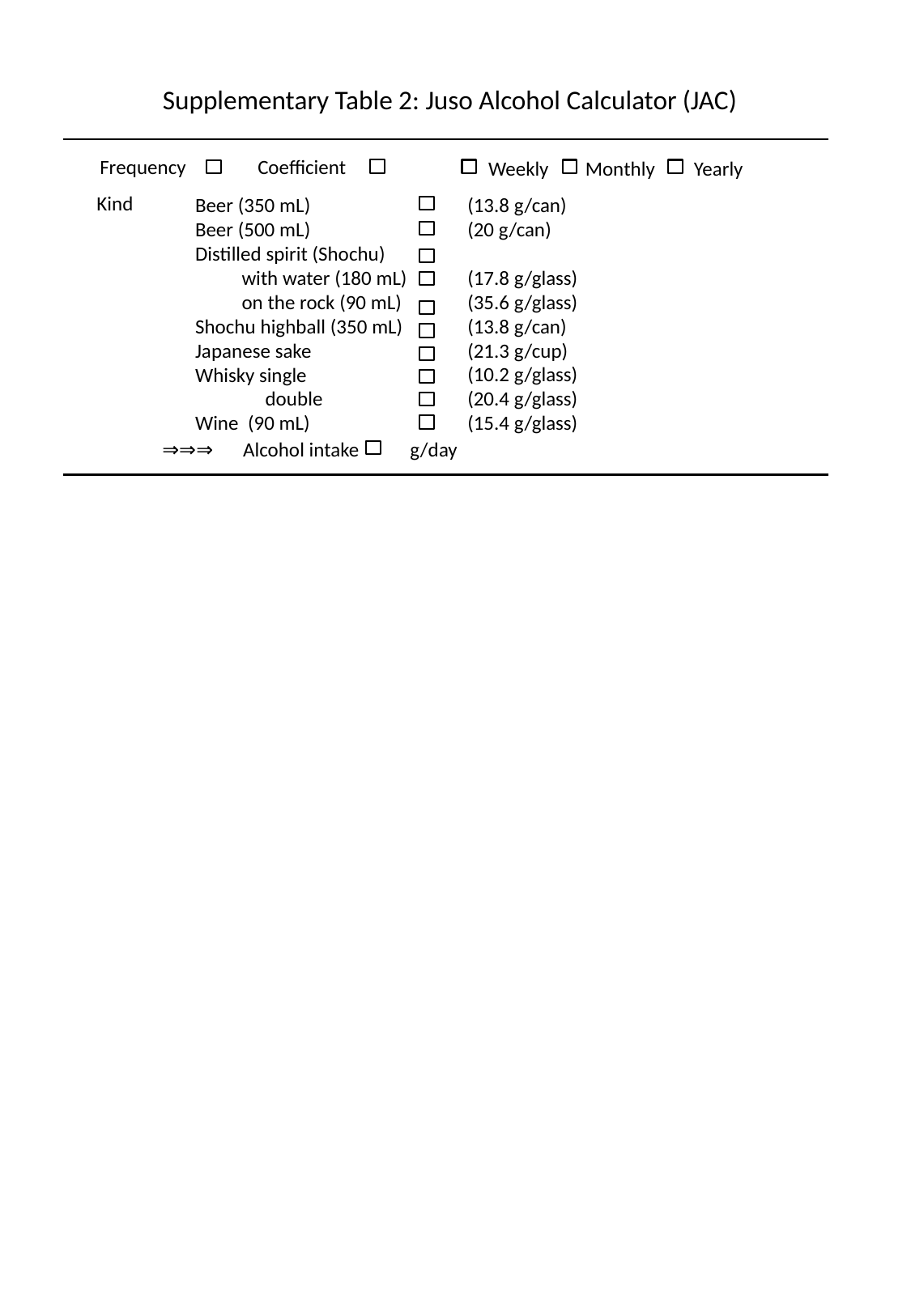

Supplementary Table 2: Juso Alcohol Calculator (JAC)
Frequency
Coefficient
Weekly
Monthly
Yearly
Kind
Beer (350 mL)
Beer (500 mL)
Distilled spirit (Shochu)
 with water (180 mL)
 on the rock (90 mL)
Shochu highball (350 mL)
Japanese sake
Whisky single
 double
Wine (90 mL)
(13.8 g/can)
(20 g/can)
(17.8 g/glass)
(35.6 g/glass)
(13.8 g/can)
(21.3 g/cup)
(10.2 g/glass)
(20.4 g/glass)
(15.4 g/glass)
⇒⇒⇒　Alcohol intake g/day
